# Plasma and whole brain cholinesterase activities in three wild bird species in Mosul, IRAQ: *In vitro* inhibition by insecticides

**DOI:** 10.2478/v10102-011-0022-x

**Published:** 2011-09

**Authors:** Ashraf S. Alias, Muna H.I. Al-Zubaidy, Yaareb J. Mousa, Fouad K. Mohammad

**Affiliations:** Department of Physiology, Biochemistry and Pharmacology, College of Veterinary Medicine, University of Mosul, P. O. Box 11136, Mosul, Iraq

**Keywords:** biomonitoring, cholinesterase, insecticides, organophosphate, wild birds

## Abstract

Plasma and brain cholinesterase activities were determined in three wild bird species to assess their exposure to organophosphate and carbamate insecticides which are used in agriculture and public health. In the present study, we used an electrometric method for measurement of cholinesterase activities in the plasma and whole brain of three indigenous wild birds commonly found in northern Iraq. The birds used were apparently healthy adults of both sexes (8 birds/species, comprising 3–5 from each sex) of quail (*Coturnix coturnix*), collard dove (*Streptopelia decaocto*) and rock dove (*Columba livia gaddi*), which were captured in Mosul, Iraq. The mean respective cholinesterase activities (Δ pH/30 minutes) in the plasma and whole brain of the birds were as follows: quail (0.96 and 0.29), collard dove (0.97and 0.82) and rock dove (1.44 and 1.42). We examined the potential susceptibility of the plasma or whole brain cholinesterases to inhibition by selected insecticides. The technique of *in vitro* cholinesterase inhibition for 10 minutes by the organophosphate insecticides dichlorvos, malathion and monocrotophos (0.5 and 1.0 µM) and the carbamate insecticide carbaryl (5 and10 µM) in the enzyme reaction mixtures showed significant inhibition of plasma and whole brain cholinesterase activities to various extents. The data further support and add to the reported cholinesterase activities determined electrometrically in wild birds in northern Iraq. The plasma and whole brain cholinesterases of the birds are highly susceptible to inhibition by organophosphate and carbamate insecticides as determined by the described electrometric method, and the results further suggest the usefulness of the method in biomonitoring wild bird cholinesterases.

## Introduction

Inhibition of plasma or brain cholinesterase activity is a biomarker endpoint of exposure of wild birds to organophosphate and carbamate insecticides (Wilson, 1999; Cocker *et al.*, 2002; Kwong, 2002; Wilson, 2005; Fildes *et al.*, 2009). Organophosphate and carbamate insecticides are extensively used in public health, veterinary practice and agriculture (Coggon, 2002; Jaga & Dharmani, 2003). These insecticides induce muscarinic, nicotinic and central nervous system toxicoses in birds by inhibition of the target enzyme cholinesterase with subsequent accumulation of acetylcholine at the nerve terminals and neuromuscular junctions (Kwong, 2002; Rusyniak & Nanagas, 2004; Wilson, 2005). Birds are naturally deficient in cholinesterase activity in erythrocytes. Therefore the diagnosis and monitoring of exposure to anticholinesterase insecticides rely on the measurement of serum or plasma and brain cholinesterase activities in wild birds (Cairns *et al.*, 1991; Burn & Leighton, 1996; Wilson, 1999; Iko *et al.*, 2002; Claudie *et al.*, 2005; Osten *et al.*, 2005; Roy *et al.*, 2005; Wilson, 2005; Fildes *et al.*, 2009). The extent of cholinesterase inhibition and intoxication with the insecticides depends on factors such as the type of the insecticide, amounts the birds are exposed to, duration of exposure, frequency of exposure, route of exposure, species variation and the degree of environmental contamination (Osweiler, 1996; Wilson *et al.*, 1998; Wilson, 1999; 2005; Wilson *et al.*, 2005).

We reported a simple electrometric method for measurement of blood or tissue cholinesterase activity in several animal species (for review see Mohammad, 2007) including the chicken (Al-Badrany & Mohammad, 2007; Mohammad *et al.*, 2008) and wild birds (Alias & Mohammad, 2005). In an initial attempt, Alias and Mohammad (2005) applied the electrometric method for measuring plasma and tissue cholinesterase activities in four indigenous wild birds (quail, large pin-tailed sand grouse, starling and rock dove) in northern Iraq (Mosul). The method of *in vitro* cholinesterase inhibition has various toxicological applications and it is used to assess the potential anticholinesterase activity of insecticides (Iyaniwura, 1990; Khan *et al.*, 1990; Karanth & Pope, 2003; Mohammad *et al.*, 2006; 2007a,b). The purpose of the present study was to further examine cholinesterase activity in wild birds (quail (*Coturnix coturnix*), collard dove (*Streptopelia decaocto*) and rock dove (*Columba livia gaddi*) in Mosul, Iraq, as well as the potential susceptibility of plasma or whole brain cholinesterases to *in vitro* inhibition by selected organophosphate (dichlorvos, malathion and monocrotophos) and carbamate (carbaryl) insecticides.

## Materials and methods

### Animals

The birds used in the present study were apparently healthy adults of both sexes of quail (*Coturnix coturnix*), collard dove (*Streptopelia decaocto*) and rock dove (*Columba livia gaddi*), which were captured in Mosul, Iraq. The birds were obtained from regions in which there were no activities of insecticide application for at least two months. The birds of each species were separately kept in captivity cages (80×60×70 cm) at about 25 °C with water and poultry feed available *ad libitum* for 3–7 days before experiments. The Scientific Committee of the College of Veterinary Medicine at the University of Mosul (Iraq) has approved the present study. All the experiments complied with institutional regulations addressing animal use, and proper attention and care were given to the birds used in this study.

Eight apparently healthy birds from each species (3–5 from each sex) were euthanized by decapitation and blood samples were collected using heparinized test tubes (Coles, 1986). Plasma was separated from erythrocytes by centrifugation at 3 000 rpm (Centurion, U.K.) for 15 min. The whole brain was excised from each bird. All plasma samples and brains were kept frozen at −20 °C pending analysis within one week.

### Electrometric method for measurement of cholinesterase activity

The whole brain was homogenized on an ice bath by a teflon homogenizer (Karl Kolb, Germany) using 25% of the maximum velocity of the homogenizer. The homogenization of the whole brain was performed in a pH 8.1 barbital-phosphate buffer solution (1.237 g sodium barbital, 0.163 g potassium dihydrogen phosphate and 35.07 g sodium chloride/L of distilled water) at 3 ml/100 mg wet weight (Mohammad, 2007; Al-Badrany & Mohammad, 2007). We determined cholinesterase activity in the plasma and brain samples by an electrometric method (Alias & Mohammad, 2005; Mohammad 2007; Al-Badrany & Mohammad, 2007). The reaction mixture of the enzyme in a 10-ml vial contained 3 ml of the pH 8.1 barbital-phosphate buffer, 0.2 ml plasma or whole brain homogenate and 3 ml of distilled water. Initial pH of the mixture (pH 1) was measured with a glass electrode using a pH meter (Hanna, Romania), then 0.10 ml of the aqueous solution of the substrate 7.5% acetylthiocholine iodide was added to the mixture which was incubated at 37 °C for 30 min. At the end of the incubation period, the pH of the reaction mixture (pH 2) was measured. The enzyme activity expressed as ΔpH/30 min was calculated as follows:

Cholinesterase activity:(ΔpH/30 min.) = (pH 1–pH 2) – Δ pH of blank.

The blank was without the plasma or brain homogenate aliquot.

### *In vitro* cholinesterase inhibition

Plasma and whole brain samples from each bird species were also used for *in vitro* cholinesterase inhibition. We used the method of inhibitor-cholinesterase incubation at 37 °C for 10 min to cause *in vitro* inhibition of plasma and whole brain cholinesterase activities of the birds (Mohammad *et al.*, 1997; 2006; 2007a,b). The commercial insecticides used were the organophosphates dichlorvos (50%, Super Nogos, Pacific Agriscience, Australia), malathion (50%, VAPCO, Jordan) and monocrotophos (40%, Green River, Italy) and the carbamate insecticide carbaryl (85%, Sociedad Anonima De Agroquimicos, Spain). The insecticides were prepared in distilled water and individually added in a volume of 0.1 ml to the reaction mixtures of the plasma or whole brain homogenate and the final reaction volume in control and inhibited samples was 6.3 ml (Mohammad *et al.*, 2007a). The final concentrations of dichlorvos, malathion and monocrotophos were 0.5 and 1.0 µM and those of the carbaryl were 5 and10 µM in the enzyme reaction mixture. Control reaction mixtures did not contain any insecticide, and they were used for measurement of base-line cholinesterase values. The reaction mixtures were incubated at 37 °C for 10 minutes and the residual cholinesterase activity in the mixtures was measured as described above. The % of inhibition of cholinesterase activity was calculated as follows:% Cholinesterase inhibition = [Cholinesterase activity (without insecticide) – Cholinesterase activity (with insecticide) / Cholinesterase activity (without insecticide)] × 100.

### Statistics

The data were presented as means ± SE and they were subjected to analysis of variance followed by the least significant difference test (Petrie & Watson, 1999). The accepted level of statistically significant difference level was at *p<*0.05.

## Results

Mean plasma cholinesterase activities of the birds in descending order were observed in the rock dove, collard dove and quail (Table 1). The plasma cholinesterase activity of the rock dove was significantly (*p<*0.05) higher than those of the other two species (Table 1). Those of the whole brain, in statistically significant descending order, were observed in the rock dove, collard dove and quail (Table 1). In the quail and collard dove, plasma cholinesterase activities were significantly higher than those of the whole brain (Table 1). In the rock dove, the difference between plasma and whole brain cholinesterase activities did not attain statistical significance level (*p>*0.05). There were no significant gender differences in plasma and whole brain cholinesterase activities of the birds.

**Table 1:** Plasma and whole brain cholinesterase activities (Δ pH/30 minutes) of apparently healthy quail (*Coturnix coturnix*), collard dove (*Streptopelia decaocto*) and rock dove (*Columba livia gaddi*) in Mosul, Iraq.

Bird species	Plasma	Whole brain
Quail	0.96±0.026 ^a^	0.29±0.023 ^a^*
Collard dove	0.97±0.031 ^a^	0.82±0.046 ^b^*
Rock dove	1.44±0.011 ^b^	1.42±0.064 ^c^

Values are mean±SE of 8 birds/species. Mean values within a column with different letters are significantly different from each other at *p<*0.05.

* Significantly different from the corresponding plasma cholinesterase activity, *p<*0.05.

Using the technique of *in vitro* cholinesterase inhibition for 10 minutes, the insecticides dichlorvos, malathion, monocrotophos and carbaryl variably and significantly inhibited plasma (9–100%) and whole brain (21–98%) cholinesterase activities (Table 2). Among the insecticides, dichlorvos caused the highest percentage of cholinesterase inhibition in the plasma of the quail (100%) and the whole brain of the rock dove (98%), (Table 2).

**Table 2:** *In vitro* inhibition of plasma and whole brain cholinesterase (ChE) activities (Δ pH/30 minutes) in wild birds by dichlorvos, malathion, monocrotophos and carbaryl.

Insecticide	Conc. (µM)	Plasma ChE	% inhib.	Whole brain ChE	% inhib.
**Quail**					
Control	0	0.96±0.026		0.29±0.023	
	0.5	0.03±0.014*	97	0.09±0.014*	69
Dichlorvos	1.0	0.0±0.0*	100	0.04±0.004*^a^	86
	0.5	0.75±0.056*	22	0.08±0.009*	72
Malathion	1.0	0.73±0.056*	24	0.10±0.025*^a^	66
	0.5	0.87±0.034*	9	0.07±0.009*	76
Monocrotophos	1.0	0.36±0.020*^a^	63	0.23±0.024*^a^	21
	5	0.35±0.020*	64	0.08±0.008*	72
Carbaryl	10	0.27±0.010*^a^	72	0.03±0.005*^a^	90
					
**Collard dove**					
Control	0	0.97±0.031		0.82±0.046	
	0.5	0.09±0.009*	89	0.05±0.009*	95
Dichlorvos	1.0	0.11±0.005*	87	0.04±0.004*	96
	0.5	0.88±0.076*	9	0.53±0.009*	35
Malathion	1.0	0.79±0.013*^a^	18	0.45±0.055*^a^	45
	0.5	0.70±0.005*	28	0.54±0.011*	34
Monocrotophos	1.0	0.37±0.025*^a^	62	0.48±0.017*^a^	41
	5	0.60±0.023*	38	0.21±0.019*	74
Carbaryl	10	0.57±0.013*^a^	41	0.13±0.011*^a^	84
					
**Rock dove**					
Control	0	1.44±0.011		1.42±0.064	
	0.5	0.04±0.009*	97	0.11±0.021*	92
Dichlorvos	1.0	0.02±0.003*	99	0.03±0.004*	98
	0.5	0.62±0.023*	57	0.18±0.028*	87
Malathion	1.0	0.47±0.009*^a^	67	0.13±0.013*	91
	0.5	1.27±0.036*	12	0.19±0.021*	87
Monocrotophos	1.0	1.09±0.008*^a^	24	0.13±0.011*	91
	5	1.26±0.013*	13	0.16±0.010*	89
Carbaryl	10	0.90±0.008*^a^	38	0.05±0.003*^a^	96

n=8 birds/ concentration group.

* Significantly different from the respective control (0 concentration), *p<*0.05.

a Significantly different from the respective lower concentration group of the insecticide, *p<*0.05.

## Discussion

The present findings further support and add to previously published findings from our lab on wild bird cholinesterase in Mosul, Iraq (Alias & Mohammad, 2005). The method we used for measurement of plasma and whole brain cholinesterase activities is an electrometric one validated for use in mammals (Mohammad, 2007) and avian species (Abass & Mohammad, 2004; Mohammad & Al-Baggou, 2005; Al-Badrany & Mohammad, 2007; Mohammad *et al.*, 2008). Determination of plasma or serum and brain cholinesterase activities in wild birds is used as a biomarker of exposure to widely used agricultural insecticides, organophosphates and carbamates (Burn & Leighton, 1996; Iko *et al.*, 2003; Osten *et al.*, 2005). It is also a major tool for diagnosing organophosphate and carbamate poisoning in birds. It is therefore important to measure wild bird cholinesterase activities periodically to assess any environmental contamination by insecticides (Hooper, 1988; Cairns *et al.*, 1991; Fossi *et al.*, 1992; Wilson & Henderson, 1992; McInnes *et al.*, 1996; Iko *et al.*, 2003; Fildes *et al.*, 2009). The present electrometric technique for measurement of cholinesterase activity was used to assess poisoning induced by organophosphate and carbamate insecticides in chickens (Abass & Mohammad, 2004; Mohammad & Al-Baggou, 2005; Al-Badrany & Mohammad, 2007; Mohammad *et al.*, 2008) and to estimate the level of total and true cholinesterase activity in the plasma of wild birds (Alias & Mohammad, 2005). True cholinesterase activity in the plasma of wild birds is usually higher than that of mammals (Wilson *et al.*, 1998; Wilson, 1999; Alias & Mohammad, 2005; Mohammad *et al.*, 2007b). It might be differentially inhibited by organophosphate or carbamate pesticides (Osweiler *et al.*, 1985; Osweiler, 1996; Wilson *et al.*, 1998; Wilson, 1999; 2005). Therefore, measuring plasma (or serum) cholinesterase activity in wild birds could give a dependable evaluation and interpretation of exposure of wild birds to anticholinesterase insecticides (Fairbrother & Bennett, 1988; Hooper, 1988; Cairns *et al.*, 1991; Fossi *et al.*, 1992; Vodela & Dalvi, 1995; McInnes *et al.*, 1996; Clegg & van Gemert, 1999; Parson *et al.*, 2000; Iko *et al.*, 2003; Fildes *et al.*, 2009).

The variations in the plasma and whole brain cholinesterase activities across the three bird species of the present study (Table 1) could be attributed to normal physiological differences found in the birds (Hill & Murray, 1987; Fairbrother & Bennett, 1988; Hooper, 1988; Fossi *et al.*, 1996; Wilson *et al.*, 1998; Wilson, 1999; Alias & Mohammad, 2005). Such differences could form the basis of differential toxicity of organophosphates and carbamates in wild birds (Fairbrother & Bennet, 1988; Hooper, 1988; Wilson *et al.*, 1998; Wilson, 1999). This notion supports the variations in the extent of *in vitro* inhibition of plasma and brain cholinesterase activities by organophosphate and carbamate insecticides (Table 2). The technique of *in vitro* cholinesterase inhibition by organophosphate and carbamate insecticides can be used to assess the potential toxicity of anticholinesterase chemicals (Iyaniwura, 1990; Khan *et al.*, 1990; Karanth & Pope, 2003; Mohammad *et al.*, 2006, 2007a b).

Organophosphate and carbamate insecticides in the avian species as well as in mammals primarily inhibit nervous system cholinesterases with subsequent development of nicotinic, muscarinic and central nervous toxicities (Anonymous, 1986a, b; Clegg & van Gemert, 1999; Wilson, 1999, 2005). However, many factors might affect the degree of cholinesterase inhibition in birds. These include the type of the insecticides, exposure dose, route and duration of exposure, species involved, toxicokinetics of the insecticide, seasonal variation, etc. (Osweiler *et al.*, 1985; Anonymous, 1986a, b; Hill & Murray, 1987; Osweiler, 1996; Wilson, 1999; Blakely & Yole, 2002; Roy *et al.*, 2005; Wilson, 2005). The *in vitro* inhibition of plasma and whole brain cholinesterase activities by the organophosphate insecticides dichlorvos, malathion, monocrotophos and by the carbamate insecticide carbaryl in the three bird species are in agreement with the reported anticholinesterase effects of these insecticides in various animal species (Anonymous, 1986a, b; Karanth & Pope, 2003; Long *et al.*, 2003; Wilson, 2005; Al-Badrany & Mohammad, 2007; Mohammad *et al.*, 2007a, b). *In vitro* cholinesterase inhibition is also a useful technique for detecting the potential anticholinesterase activity of chemicals (Iyaniwura, 1990; Khan *et al.*, 1990; Karanth & Pope, 2003; Mohammad *et al.*, 2006, 2007a, b).

In conclusion, the present study reports the values of plasma and whole brain cholinesterase activities in three indigenous wild birds (quail, collard dove and rock dove) in Mosul, Iraq, as determined by an electrometric method. The present findings are indicative of the sensitivity of the described method in detecting cholinesterase inhibition caused by organophosphates or carbamates. As these enzymes are potential targets for anticholinesterase insecticides, their activities could be routinely measured using the described electrometric method to assess exposure of wild birds to agricultural insecticides polluting the environment.
